# Performance Analysis of Keypoints Detection and Description Algorithms for Stereo Vision Based Odometry

**DOI:** 10.3390/s25196129

**Published:** 2025-10-03

**Authors:** Sebastian Budzan, Roman Wyżgolik, Michał Lysko

**Affiliations:** Department of Measurements and Control Systems, Silesian University of Technology, Akademicka 16, 44-100 Gliwice, Poland; roman.wyzgolik@polsl.pl (R.W.); michlys910@student.polsl.pl (M.L.)

**Keywords:** keypoint detection, keypoint description, GFTT, ORB, BRISK, KAZE, visual odometry, SLAM

## Abstract

This paper presents a comprehensive evaluation of keypoint detection and description algorithms for stereo vision-based odometry in dynamic environments. Five widely used methods—FAST, GFTT, ORB, BRISK, and KAZE—were analyzed in terms of detection accuracy, robustness to image distortions, computational efficiency, and suitability for embedded systems. Using the KITTI dataset, the study assessed the influence of image resolution, noise, blur, and contrast variations on keypoint performance. The matching quality between stereo image pairs and across consecutive frames was also examined, with particular attention to drift—cumulative trajectory error—during motion estimation. The results show that while FAST and ORB detect the highest number of keypoints, GFTT offers the best balance between matching quality and processing time. KAZE provides high robustness but at the cost of computational load. The findings highlight the trade-offs between speed, accuracy, and resilience to environmental changes, offering practical guidance for selecting keypoint algorithms in real-time stereo visual odometry systems. The study concludes that GFTT is the most suitable method for trajectory estimation in dynamic, real-world conditions.

## 1. Introduction

Keypoints descriptors are numerical representations of local image features that enable them to be identified and matched unambiguously between different images of the same scene. In recent years, the task of keypoint detection and description has been an active area of research. The initial main fields of application for keypoint detection and description were image representation [1,2], image retrieval and classification [3], image matching [4], 3D scene reconstruction [5], object recognition [6], object tracking [7,8], texture classification [9] and biometrics systems [10,11].

The rapid development of technology in the field of mobile robots and autonomous vehicles has made it necessary to search for effective localization and tracking methods. A variety of methods for locating mobile robots have been developed, primarily relying on GPS signals for outdoor applications. Indoor solutions use incremental or inertial odometry alongside internal IMU measurement units, RFID tags, UWB or Bluetooth [12]. Last years, there has been an increasing demand for efficient, real-time localization solutions based on computer vision that can operate effectively in dynamic and changing environments. One of the most promising methods of localization for mobile robots is visual odometry [13] with mono or stereo camera. Visual odometry is a fundamental method for estimating motion, which includes keypoint detection, establishing correspondence, and estimating pose. Generally, the visual odometry algorithm with a stereo camera system estimates motion by using depth information of the observed scene by calculating the relative distance of objects from the camera. This process involves acquiring synchronized images from two spatially separated cameras, which enables the reconstruction of three-dimensional spatial information through the analysis of discrepancies. By comparing matching features in the left and right images, the algorithm can measure the difference in their positions—known as disparity—which allows it to reconstruct the scene in three dimensions and determine the depth of each object in the scene. Thanks to stereovision, the translation and rotation of objects in an image can be estimated, as well as the camera’s movement parameters relative to its environment. Therefore, accurate depth estimation is essential for enabling motion tracking and local environment mapping. Conversely, the accuracy of depth estimation depends directly on the process of keypoints detection.

Visual odometry is the foundation of SLAM (Simultaneous Localization and Mapping) algorithms, which enable the concurrent construction of an environmental map and the estimation of a mobile robot’s position and trajectory within a global reference frame. These algorithms typically rely on successive images captured as the robot—most often an Autonomous Mobile Robot (AMR)—navigates through its surroundings. In publication [14], the authors conducted an in-depth analysis of various SLAM methods, with particular emphasis on vision-based approaches. They demonstrated the superior capabilities of these methods compared to alternative localization techniques and identified several critical components that influence the effectiveness of SLAM systems: keypoint detection and description, loop closure mechanisms, map updating processes, position estimation, keypoint tracking, and final object motion trajectory tracking.

Over the past decades, numerous SLAM algorithms leveraging keypoints have emerged, with ORB-SLAM, ORB-SLAM2, and ORB-SLAM3 [15] standing out for their use of the ORB (Oriented FAST and Rotated BRIEF) algorithm to detect and describe distinctive keypoints, enabling highly accurate real-time localization and mapping. Another notable method is PTAM (Parallel Tracking and Mapping) [16], which separates tracking and mapping into parallel processes, making it particularly effective in confined environments. In RGB-D SLAM algorithm [17] keypoints are extracted from RGB images and spatially localized using depth data, facilitating robust 3D mapping. The GraphSLAM algorithm [18], which centers on graph-based optimization, can utilize keypoints as input features in its vision-based implementations.

Keypoint descriptors are central to these systems because they allow for consistent environmental mapping based on image sequences. It is important to distinguish between feature detectors, which identify and locate points of high informational value in an image, and feature descriptors, which transform those points into numerical vectors. These representations facilitate the comparison and matching of points across images, ensuring continuity in position estimation and map updates within SLAM frameworks. Keypoints are divided into three main types: binary, gradient, and hybrid [19,20,21,22]. Binary descriptors, such as ORB, BRISK (Binary Robust Invariant Scalable Keypoints), FAST (Features from Accelerated Segment Test) and BRIEF (Binary Robust Independent Elementary Features), use local comparisons of pixel intensities, offering fast matching thanks to Hamming distance and low memory requirements. Gradient-based descriptors, such as SIFT (Scale-Invariant Feature Transform), SURF (Speeded-Up Robust Features), and KAZE (KAnizy Zernike Descriptors), analyze histograms of gradient directions and magnitudes, providing greater robustness to lighting changes at the cost of higher computational complexity. Hybrid algorithms, such as AKAZE and KAZE, combine the advantages of both approaches, offering a compromise between speed and accuracy, often available in binary and floating-point versions.

Researchers are conducting scientific research on using neural networks to detect and describe characteristic points. The first method was the LIFT (Learned Invariant Feature Transform) [23] method. In this method, the authors proposed combining keypoint detection, orientation estimation, and descriptor calculation into one process. Training begins with learning the descriptor module, which informs training of the orientation estimator. Finally, the detector is trained using the previously learned descriptor and orientation estimator, with gradients propagated through the entire network. During inference, the detector operates independently across the full image and scale space. Meanwhile, the orientation estimator and descriptor are applied only to selected keypoints. This method yields high-quality keypoints but requires more computation time. One popular method is SuperPoint [24]. SuperPoint simultaneously detects characteristic points and generates their descriptors. In the initial stage, the method uses synthetic images containing simple objects, such as cubes, quads, lines, and stars. Then, SuperPoint adapts to real images via homographic adaptation to generate pseudo-ground truth interest points. Due to its use of GPU systems, it is characterized by considerable speed, as well as resistance to changes in lighting, rotation, scale and interference. On the other hand, it requires fine tuning when adapting it to a specific task. Next method, LF-Net [25] consists of two main components: a detector network that produces a heatmap indicating the probability of keypoint locations and a descriptor network that generates floating-point descriptors for each detected point. During training, LF-Net uses a differentiable warping mechanism to align keypoints between image pairs, evaluating their repeatability and matching accuracy. The network is optimized to maximize correct matches-inliers and minimize incorrect ones–outliers. Like previous methods, the D2-Net [26] method uses a convolutional neural network (CNN) to generate a dense feature map of the input image. These feature maps are then used to calculate descriptors and detect keypoints, which are the local maxima of the feature maps. This approach enabled the authors to identify geometrically stable points, achieving a high degree of match between the keypoint and the descriptor. The proposed method is characterized by reduced memory load and similar detection efficiency. In [27] authors proposed R2D2 method, which uses a CNN to generate a dense feature map of the input image. These feature maps are then used to calculate descriptors and detect keypoints, which are the local maxima of the feature maps. This approach enabled the authors to identify geometrically stable points, achieving a high degree of match between the keypoint and the descriptor. The proposed method is characterized by reduced memory load and similar detection efficiency. One advantage of the method is the generation of high-quality keypoints. However, its disadvantage is that it has a greater computational load compared to the SuperPoint method.

Ideal feature detectors exhibit specific properties, such as robustness to noise and transformations, repeatability across varying conditions, high localization accuracy, general applicability, computational efficiency, and sufficient feature density. Ideal features are consistently detectable, carry discriminative information, and are precisely localized within the image domain.

The dynamic growth in recent years of the capabilities of embedded systems used in the control of mobile robots has enabled the practical implementation of many methods that were originally used in computer vision. The research and methods proposed in this paper enable the evaluation of the usefulness of selected methods for detecting and describing keypoints in practical applications, such as visual odometry. The research presented in this article has been conducted using classic methods that are applicable and implementable in commercial visual odometry solutions based on the embedded systems, which have limited resources compared to systems based on PC or notebooks. The following methods were selected: FAST (Features from Accelerated Segment Test), GFTT (Good Features to Track), ORB, BRISK, and KAZE. The authors’ research focused on evaluating the aforementioned methods for use in visual odometry scheme. The choice of method is crucial for effectively using the system in real-world conditions because the selection and parameterization determine the number and quality of position tracking and affect the maximum speed of the object’s movement and the dynamics of its direction changes. For example, this involves selecting the minimum number of keypoints required in relation to processing time. The accuracy of location and the ability to track the object over time are also affected by an insufficient number of keypoints in the object’s surroundings due to the lack of connection between landmarks in successive frames. In SLAM methods, such a connection is essential. Unlike most contemporary SLAM algorithms, which rely on the fundamental assumption of a static environment composed of rigid, non-moving objects, our research specifically addresses the challenges posed by dynamic scenes. In such environments, where objects may move independently and lighting may vary, achieving robust and accurate pose estimation and localization is significantly more complex. The dynamically changing environment around the object directly affects the cumulative driving error and the repeatability of position estimation on the map, particularly when there are rapid changes in the field of view of the camera, such as turns in the driving path.

The object’s dynamically changing environment directly impacts the cumulative driving error and repeatability of position estimation on the map. The disadvantage of all trajectory determination methods based on visual odometry is the accumulation of estimation errors in the trajectory. Subsequent positions are determined based on previous ones, causing the accumulation of errors made in subsequent steps. This systematic deviation of the estimated position from the actual position, caused by the accumulation of errors, is called drift [28]. Clearly, minimizing drift directly increases location accuracy. The basic method of eliminating drift is improving the quality of detected keypoints and correctly matching them between stereo images from the right and left cameras, as well as between successive image frames obtained during the trajectory study. This requires selecting the minimum number of feature points necessary for processing. However, in dynamic scenes, unlike static ones, which mainly interest researchers, disturbances in object features can easily cause trajectory drift and system failure. Other hand, the number of detected keypoints affects processing time and, consequently, computational load. Keypoint detection and descriptor creation should take place quickly enough to prevent the scene in front of the vehicle from changing so much that it becomes impossible to estimate the vehicle’s trajectory due to a lack of connection between neighboring frames via the detected keypoints.

**Figure 1:**
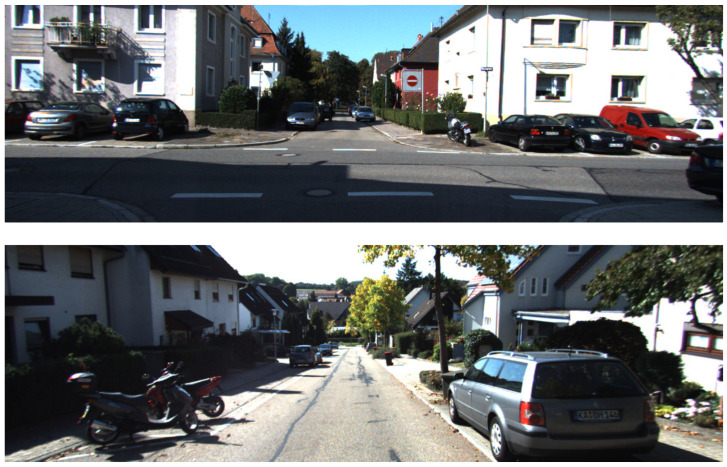
Selected sample images from KITTI dataset.

The evaluation of visual odometry detectors and descriptors involves a multifaceted set of criteria rather than a single performance metric. These criteria encompass the quantity and quality of detected keypoints, the detection methodology, sensitivity to motion dynamics, and repeatability—where the latter is particularly critical for reliable odometry. To address these aspects, the research presented in this paper utilizes the KITTI stereo image dataset [29], which comprises real-world driving scenarios captured under natural conditions (Figure 1). A comprehensive analysis was conducted, assessing parameters such as the number of detected points, image resolution, and image quality factors including noise, distortion, and contrast variability. The evaluation also considered diverse scene types, such as road intersections, traffic environments, straight and empty roads, and urban structures. The impact of various detection methods on stereo image point matching was systematically analyzed. Finally, the influence of feature selection techniques on stereo visual odometry was quantified by estimating drift across image sequences processed using the respective methods.

## 2. Materials and Methods

### 2.1. Selected Methods of Keypoints Detection and Description with Visual Odometry Algorithm

The description of keypoints clearly indicates that they should convey image information in a way that allows them to be identified regardless of orientation or scale. As previously mentioned, keypoints only contain location information, which does not ensure adaptation to distortions. To address this, keypoints are supplemented with a description. This description concerns the surroundings of a keypoint and allows for its unambiguous identification, enabling its location regardless of the image’s orientation or scale.

The FAST method [30] is a highly effective feature point detector that is particularly useful in real-time applications. For this reason, the authors focused primarily on the algorithm’s performance. It is characterized by its ability to quickly detect corners through the analysis of pixel neighborhoods in a 16-pixel Bresenham circle. For each pixel, an intensity test is performed to determine if the pixel on the circle is brighter or darker than the center pixel, given a known threshold value. Any point that meets this criterion is classified as a keypoint. The authors also proposed modifying the algorithm by selecting pixels numbered 1, 9, 5, and 13, which significantly reduced the number of points analyzed. This method offers two key advantages: efficiency and scalability. These advantages stem from its applicability to both CPUs and GPUs. However, due to its lack of invariance to rotation and scale, it is usually employed in conjunction with other descriptors. A significant disadvantage is that it tests all pixels in the image, resulting in the detection and selection of numerous neighboring feature points.

Another efficient method is GFTT [31]. As the authors point out, it identifies patterns suitable for tracking. It draws inspiration from methods of detecting motion in images. For each pixel, intensity gradients are calculated, and then a Harris matrix is applied. The Harris matrix contains information about local changes in intensity. After determining the matrix’s eigenvalues, the Shi-Tomasi criterion is applied to identify corners. Among corner detection methods, GFTT is characterized by the greatest stability and selection of higher-quality keypoints for tracking. However, GFTT has several limitations, including a lack of scale invariance, sensitivity to noise, a lack of its own descriptors, and limited repeatability.

The next important method under investigation is ORB [20], which combines the FAST algorithm, used for rapidly detecting points in an image, with the BRIEF algorithm, used for generating binary descriptors. Only stable pixels identified by FAST are selected via Harris matrix analysis in this method. Then, an intensity moment analysis is performed to obtain a rotation vector that guarantees rotation invariance. This method is resistant to noise and much faster than SIFT/SURF methods. However, it is sensitive to changes in lighting and lacks scale invariance, which becomes apparent in more complex scenes.

Another method used during the experiments was BRISK [21] Similarly to ORB, BRISK detects keypoints and describes their features simultaneously. It uses a modified FAST algorithm for keypoint detection and a multiscale image pyramid with octaves of different resolutions, as well as an inter-octave pyramid to achieve higher sampling density. Similarly to the FAST method, BRISK performs pixel classification based on the neighborhood of the center pixel and takes images of different scales into account. BRISK implements a mechanism that resists orientation changes when describing only keypoints. This means that keypoints without a descriptor lack invariance. Thus, BRISK is rotation- and scale-invariant, making it significantly faster than SIFT and SURF, though slower than ORB. However, the method is sensitive to noise, especially in lower-quality images.

The KAZE method [22], which operates on a nonlinear scale, has also been used in the presented research. This contrasts with the linear Gaussian scale space used in SOFT and SURF, which blurs important details and merges similar structures. KAZE detects keypoints as local maxima of the Hessian matrix determinant. This allows for the precise identification of important image structures. These points are described using the G-SURF descriptor, which is an extension of the SURF algorithm adapted to work in nonlinear scale space. The KAZE method generates high-quality keypoints, is resistant to noise and interference, and exhibits rotational and scale invariance. Its biggest drawbacks are its very high computational requirements and the non-binary nature of the descriptors.

The parameters of the selected methods were set to their default values. The OpenCV library implementation of these methods was used. Table 1 lists the most important parameters. As the article provides a general comparison of methods, we used the also the default value of other parameters. For example, for K-NN, we assumed K = 2; for RANSAC, we assumed a threshold of 31; and for PnP, we used octaves of 3 (BRISK) and 4 (KAZE), set upright to false (KAZE) and set the score type to HARRIS_SCORE (ORB).

We evaluated visual odometry using keypoint detection and description methods in conjunction with stereo visual odometry. This method estimates the position and rotation of a mobile robot in each new stereo frame. The algorithm performs the following steps:

1. Epipolar rectification of the stereo images to facilitate point matching by transforming the images according to epipolar lines.
2. Detection of keypoints in the left image using one of the tested keypoint detection methods.
3. Description and matching of points in the right image using triangulation. This involves finding corresponding points in the right image and calculating their 3D position relative to the current frame.
4. Matching points between consecutive frames of the sequence. Keypoints are tracked from the current frame relative to the previous one. Matching was performed using the K-Nearest Neighbor Matching method and selected metrics. This procedure is performed by default for the left image.
5. Camera movement estimation using PnP + RANSAC algorithms. At this stage, the transformation (rotation + translation) between successive frames is calculated based on the matched 3D points, with simultaneous rejection of outliers.

### 2.2. Dataset

The research was conducted using the KITTI dataset, developed as a benchmark for visual odometry, SLAM, and optical flow tasks, except the research on image resolution and influence of image quality on the selection of keypoints (Section 3.1.2 and Section 3.1.3), which has been carried out on own images. The dataset contains sensor data recorded as a test vehicle traveled around the city and its surroundings. The acquisition system consists of stereo cameras (left and right), a Velodyne HDL-64E lidar system, a GPS system, and an IMU unit. These components provide reference data (ground truth) and assist with localization and calibration. The KITTI dataset is divided into thematic subsets, including visual odometry, stereovision, object detection, semantic segmentation, and object tracking. Particular attention should be paid to the visual odometry subset, which contains 22 sequences of stereo images with a resolution of 1241 × 376 pixels. Eleven sequences have a reference trajectory, and the remaining eleven are intended for testing algorithms without access to ground truth. Each sequence contains images from both cameras, calibration files (camera matrices and stereo parameters), and, optionally, lidar and GPS/IMU data. The KITTI dataset allows for a thorough evaluation of visual odometry algorithms. It does so by estimating the vehicle’s trajectory based on stereo images. Then, it compares the results with the reference trajectory. Finally, it evaluates feature matching, motion estimation, and trajectory optimization.

## 3. Results and Discussion

A series of experiments were conducted to evaluate the influence of feature point selection methods on the performance of stereo visual odometry algorithms, and to identify the most effective approach. The evaluation focused on the following key metrics:

- Processing time;
- Estimation accuracy;
- Robustness to errors and generalization capability.

These criteria are essential for the practical deployment of feature selection techniques in trajectory estimation under real-world conditions. All experiments were performed on a consistent dataset, under identical conditions, and using the same hardware configuration.

The application implementing the algorithms was compiled and executed on a machine equipped with an Intel(R) Core(TM) i5-7300HQ CPU @ 2.50 GHz, 8 GB RAM, and an NVIDIA GeForce GTX 1050 GPU (Intel, Santa Clara, CA, USA; NVIDIA, Santa Clara, CA, USA).

### 3.1. Keypoints Identification

In order to evaluate the performance of various keypoint detection algorithms, a series of experiments were conducted on original images as well as images subjected to different types of distortions: noise, blur, geometric distortion.

#### 3.1.1. Number of Detected Keypoints

This experiment was conducted on original images from KITTI Vision Benchmark Suite. The purpose was to evaluate the number of keypoints detected by selected methods across different types of images.

Six different image pairs were selected, each representing a unique scene commonly encountered in the road environment. These scenes included: an intersection, a traffic jam, an empty road, a straight dirt road, a road surrounded by buildings, and a solitary building along a road. Each keypoint detection method, described in this article, was applied to all selected scenes.

**Figure 2:**
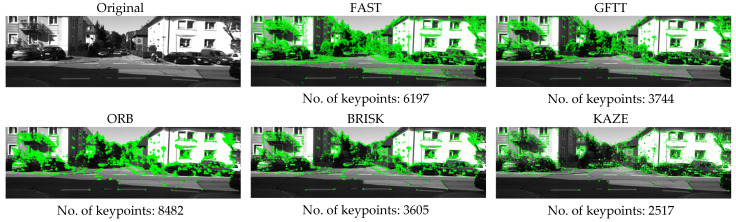
Visualization of keypoints detection for selected methods on a sample image from the selected set.

Figure 2 shows an example of examining the number of keypoints detected for the left image in the intersection scene, along with the visualization of points for each method. The results of the conducted study of the number of keypoints detected for selected methods and scenes are summarized in Table 2 and Table 3. It contains the results achieved by individual methods for the right and left images for each of the tested scenes.

Analysis of the results of the study shows that, in line with the literature, the FAST method and the ORB method based on it detect the largest number of feature points, regardless of the scene being examined.

#### 3.1.2. The Influence of Image Resolution

We are interested in implementing a stereovision odometry algorithm in real-time systems, primarily embedded systems, characterized by limited computational and memory resources. Therefore, efficient processing of subsequent image frames is crucial.

High-resolution images, despite containing more information useful for visual analysis, generate significant computational overhead, which can negatively impact algorithm performance. To reduce processing time, a common practice is to scale images to lower resolutions.

Reducing image resolution, however, leads to the loss of some spatial information, which can impact the quality of local feature extraction, particularly feature points. To analyze the impact of image resolution on feature point detection efficiency, experiments were conducted involving scaling selected images to typical resolutions used in digital image processing. The aim of the research was to determine the relationship between image resolution and the number and quality of feature points detected by selected detection algorithms.

**Figure 3:**
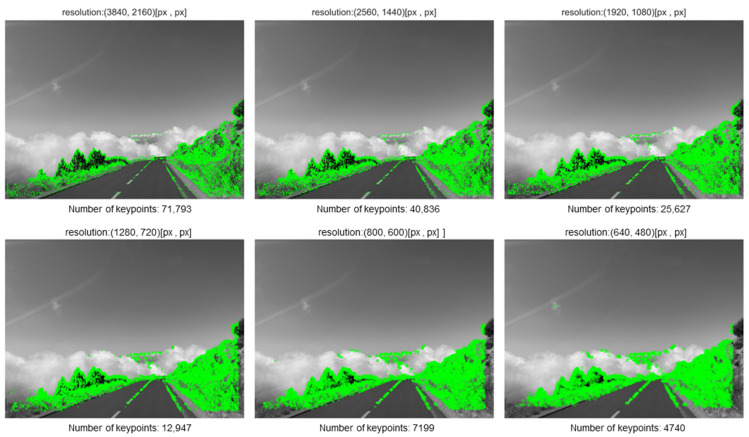
A set of images for selected resolutions with visualization of keypoints for the ORB method.

**Figure 4:**
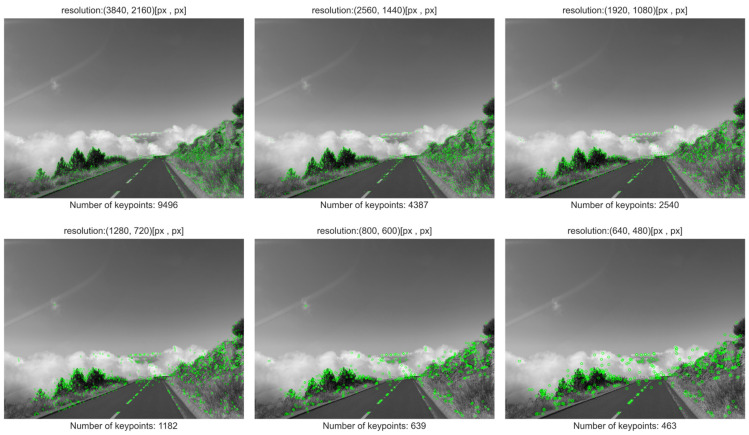
A set of images for selected resolutions with visualization of keypoints for the KAZE method.

Figure 3 and Figure 4 present the results for two out of the five evaluated feature detection methods, specifically those yielding the highest and lowest number of detected keypoints, respectively. Figure 3 illustrates the impact of pixel count (image resolution) on the number of keypoints detected by the ORB algorithm, which demonstrated the highest detection rate in the conducted experiments.

Visual analysis of the results reveals that many keypoints detected across different resolutions exhibit spatial consistency—that is, keypoints identified in lower-resolution images often coincide with those found in higher-resolution counterparts. A similar phenomenon is observed in Figure 4, which shows the results for the method with the lowest number of detected keypoints.

It is worth noting, however, that in the lowest-resolution images, additional keypoints emerge that are not present in higher-resolution versions. The appearance of these points is likely attributable to artifacts introduced during the image downsampling process, which can alter local texture and gradient characteristics, thereby influencing the behavior of feature detection algorithms. This effect is not strictly correlated with the sampling density of low-resolution images but may arise from the specific interpolation or resampling techniques applied during preprocessing.

Therefore, when designing systems that rely on keypoint detection—particularly in scenarios involving variable image resolutions—it is essential to account for the potential impact of downsampling artifacts on detection accuracy and consistency.

**Figure 5:**
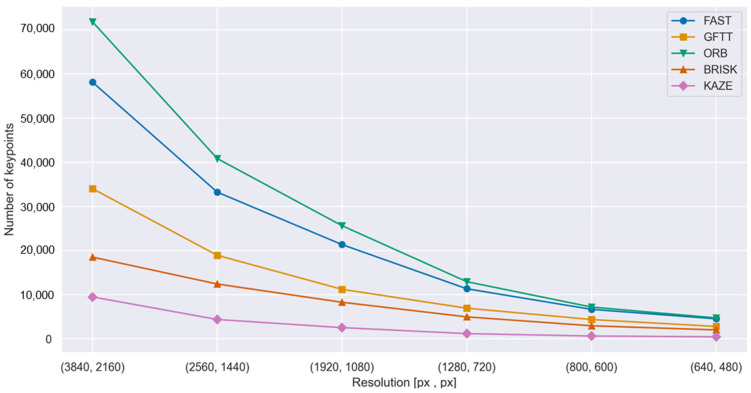
Graph of the dependence of the number of keypoints on the image resolution.

Aggregate results of the study on the impact of image resolution on the number of detected keypoints are presented in Figure 5 and summarized in Table 4. As illustrated in the graph, the observed trend is consistent across all evaluated feature detection methods: reducing image resolution leads to a decrease in the number of detected keypoints.

A more detailed analysis reveals that the magnitude of this reduction is method-dependent. Specifically, algorithms that inherently detect fewer keypoints exhibit a less pronounced decline in response to resolution reduction. Furthermore, the relative proportion of detected keypoints between methods remains approximately constant across different resolutions.

Among the tested algorithms, ORB consistently yields the highest number of keypoints, followed by FAST. In contrast, BRISK and KAZE produce the lowest counts, while GFTT occupies an intermediate position in terms of detection density.

The flattening of the curve at lower resolutions supports the hypothesis that certain keypoints exhibit resolution invariance—that is, they persist across multiple scales of the same image. This suggests the presence of structurally stable features that remain detectable regardless of pixel density, which may be particularly relevant in applications involving multi-scale image analysis or resource-constrained environments.

#### 3.1.3. The Influence of Image Quality on the Selection of Keypoints

An additional experiment was conducted to evaluate the robustness of the selected keypoint detection methods under various types of image degradation. For consistency with previous tests, an image depicting a road scene with adjacent roadside elements was selected as the test input. The following disturbances were introduced into the image:

- Additive impulsive salt and pepper noise, 30%;
- Blurring;
- Barrel distortion;
- Contrast adjustment—including both contrast enhancement and reduction to reflect varying lighting conditions.

**Figure 6:**
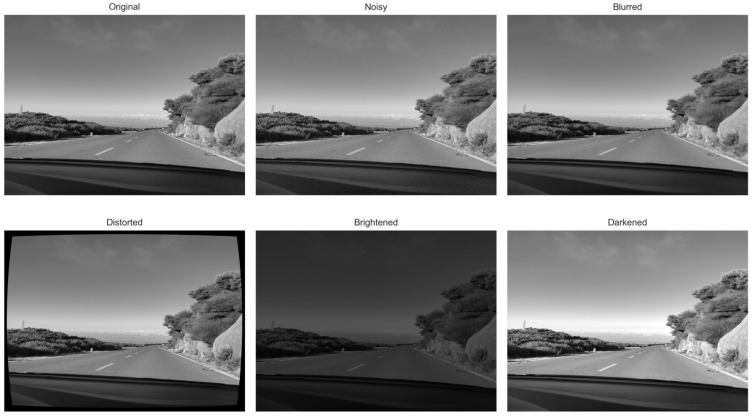
Original image along with its modifications containing introduced distortions.

**Figure 7:**
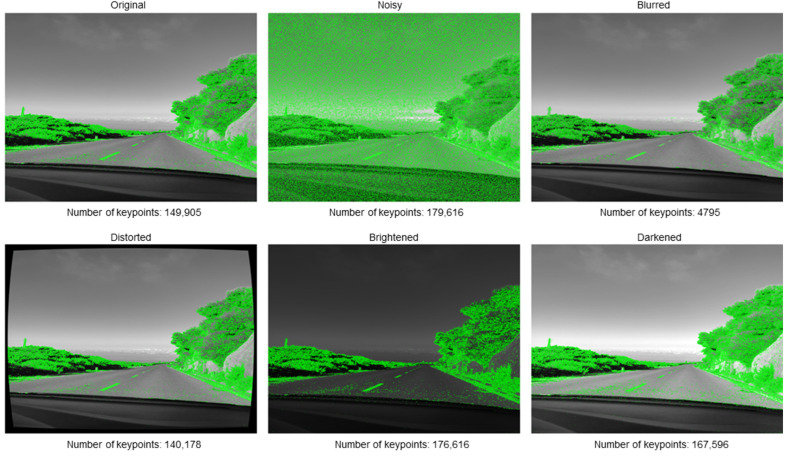
GFTT keypoints detection results in original and distorted images.

The selected image, as well as the images created by introducing distortions, are presented in Figure 6. The efficacy of the keypoint selection methods was then examined on a set of images. An example with visualization for GFTT is shown in Figure 7. The analysis of these disturbances enables the determination of their respective impacts. GFTT was selected as a representative example due to the fact that it yielded the most optimal results when compared to other examples that were tested. Even a cursory examination of the visualizations reveals the substantial impact of each disturbance.

It is imperative to pay particular attention to the outcome of the selection of keypoints in the image with impulsive noise. This result demonstrates that this type of image disturbance hinders the search for keypoints. It is evident that the disturbance exerts no direct influence on the number of keypoints located in closer proximity to the image center through the mechanism of image distortion. Conversely, points situated at the periphery of the frame become undetectable. However, such interference may result in greater interference in subsequent steps of the stereo odometry algorithm, such as triangulation. The investigation of image blurring as a form of interference has revealed its substantial impact on the identification of keypoints, as evidenced by a decline in the number of detected points. Conversely, contrast is not a factor that exerts significant influence on the GFTT method.

**Figure 8:**
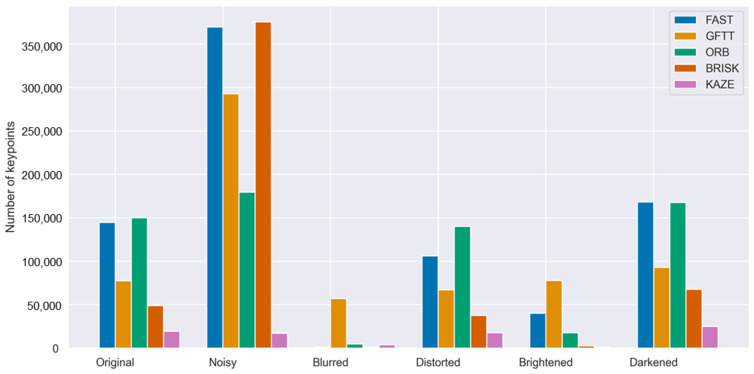
Number of keypoints detected in original and distorted images for all analyzed methods.

The bar chart in Figure 8 presents the results of the study for all of the selected methods. In the study of the impact of image disturbances on the method of selecting keypoints, the GFTT method was found to produce the most stable results, as previously mentioned. The number of keypoints was the most stable for all disturbances and the closest to the original image. The FAST method demonstrated the most significant variations in the number of points in this study. The results of the study have been compiled in Table 5, which clearly demonstrates that all methods are hindered in their usefulness by the presence of noise and blurring in the image. This phenomenon is influenced by the keypoint determination algorithm employed in the tested methods, which is based on pixel analysis of the image.

#### 3.1.4. Keypoints Matching in Stereo Vision Images

In selecting a keypoint selection method, it is imperative to consider the location at which they will be utilized. In this instance, the aforementioned methodology is known as the stereovision odometry algorithm. Consequently, the criteria previously outlined, in addition to processing speed and odometry accuracy, which will be addressed subsequently in this article, must be given due consideration. This section delineates the process of matching stereo image pairs by employing the evaluated methodologies. For the purposes of this study, the two most widely utilized keypoint descriptors, ORB and BRISK, were selected for analysis. Images comes from KITTI Vision Benchmark Suite.

**Figure 9:**
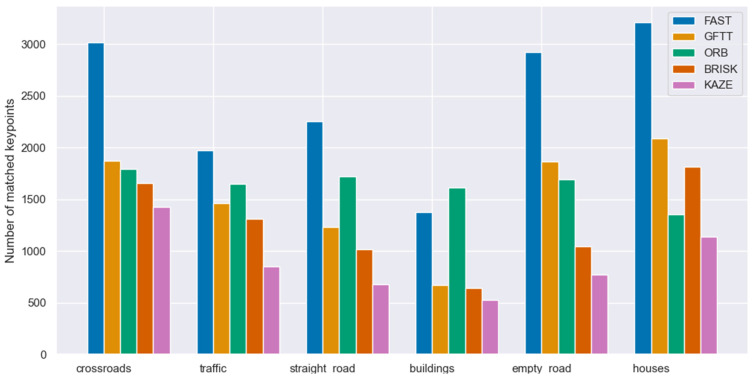
Number of matched keypoints in stereovision images with ORB descriptor.

**Figure 10:**
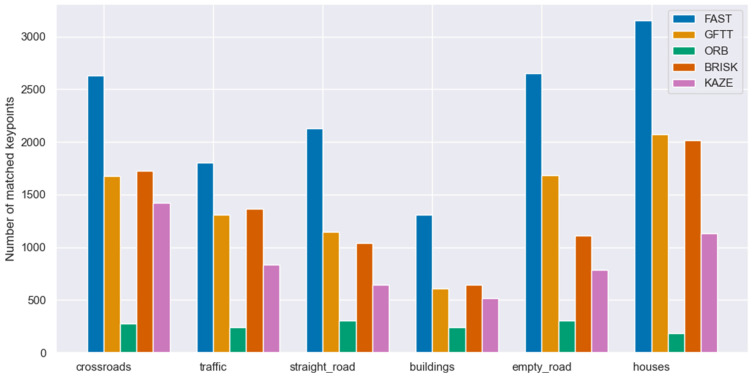
Number of matched keypoints in stereovision images with BRISK descriptor.

The application of descriptors to the designated keypoints, followed by matching using the K-Nearest Neighbor Matching method, results in a set of keypoints that are unique to the pair of stereovision images representing a given scene. Figure 9 presents the matching results for a selection of scenes with ORB descriptor while Figure 10 with BRISK descriptor. The results obtained from this study demonstrate a direct correlation between the number of matched points and the examined scene. However, as was the case with keypoint detection, the greater the number of points detected, the more points are matched.

The subsequent number of matched keypoints in the images should be verified for their usefulness. A high number of matched keypoints may be due to a high number of detected keypoints in both stereo images, but this is not necessarily indicative of quality. In order to achieve this objective, an examination was conducted of the proportion of keypoints detected in the image that were matched. In the present study, the ORB descriptor was utilized. The results presented in Table 6 demonstrate the proportion of detected keypoints in both images that were matched by each of the selected methods.

In consideration of the findings, it can be deduced that the entirety of the selected methodologies demonstrate a matched-to-detected point ratio of 58%. This indicates that a considerable proportion of the detected points will not be subjected to subsequent analysis within the framework of the stereo odometry algorithm.

The GFTT method has been demonstrated to have the most optimal match-to-detection ratio, with a result of 61.5%. The results presented in the table have been calculated as the mean value for the set of tested scenes. The study concluded that the number of landmarks is not the only factor to be considered; the quality and uniqueness of these landmarks within the context of a given scene is also important.

The final part of this study examines the keypoint processing time, using the keypoint selection method, the ORB descriptor, and the K-Nearest Neighbor matching method. As outlined in Table 7, a summary of the mean times and total points achieved for each of the test scenes is provided, averaged across all selected keypoint detection methods.

An analysis of the mean point detection time in the image reveals that the FAST method exhibits the shortest detection time, while the KAZE method demonstrates the longest detection time. In the following step, the mean number of detected keypoints is examined in order to ascertain the ratio of the number of detected keypoints during the processing time. This ratio is represented by the third row of the analyzed table. This parameter demonstrates that FAST, in accordance with the extant literature, is capable of detecting a high number of feature points with great rapidity. However, as demonstrated in previous research, it can be concluded that the quality of detected points is also important, not just their number. The subsequent row in the table presents the mean number of matches for a given method. The subsequent step concerns the processing time for the feature point matching method. The GFTT and KAZE methods are worthy of particular note, as they demonstrated the most favorable performance in this respect, with an average time of 5 ms. However, the FAST method has the worst matching time, which is likely due to the poor quality of the feature points selected with this method. These points are not unique in the scene.

A detailed analysis of the ratio of the number of matched points to the processing time indicates that the GFTT method produces the most accurate matches in the shortest time, which is its clear advantage. To conclude this study, it is imperative to emphasize the significance of contemplating the point processing speed of the examined methodologies in conjunction with the quantity of correspondingly identified feature points. This approach provides a broader perspective on the analyzed methods, allowing us to recognize the advantages of methods such as GFTT and to align with the FAST method’s authors regarding their assumptions.

### 3.2. The Influence of Feature Point Selection Methods on Stereovision Odometry

The subsequent stage of the research involved the examination of the impact of keypoint selection methods on trajectory estimation results in the stereo odometry algorithm. In accordance with the results of this research, it will be possible to assess such parameters as processing time, accuracy, and the suitability of the algorithm for application.

#### 3.2.1. Trajectory Estimation

In the course of the accuracy assessment, a series of stereo images were selected from the designated test set. The images depicted a vehicle in motion through an urban environment.

**Figure 11:**
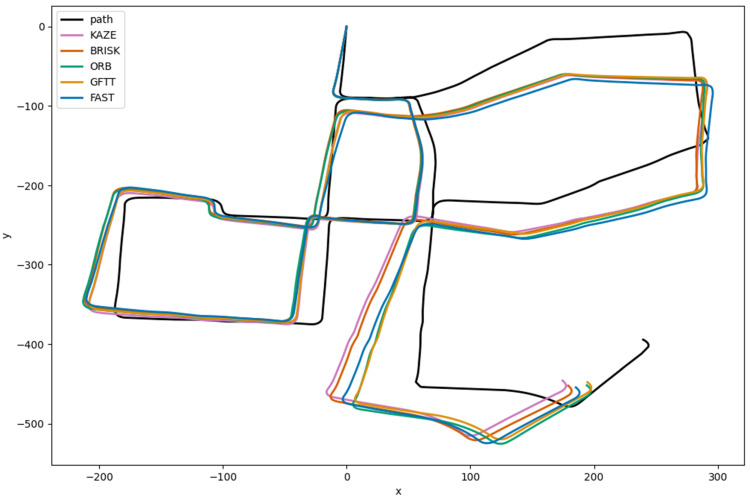
Visualization of estimated routes for the tested methods in the stereovision odometry algorithm along with the original vehicle route; x and y axes are scaled in meters.

The implemented stereo odometry algorithm was tested on this sequence. The results of the test provided values that represented the camera’s position on the vehicle, expressed as coordinates [x, y] on a two-dimensional map. As illustrated in Figure 11, a visual representation of the trajectory estimates for all of the methods that were examined is provided.

The initial vehicle trajectory, calculated on the basis of the provided GPS data, is also delineated by a black line. The trajectory estimate for the initial pair of stereo images and the actual route are initiated at point [0.0, 0.0].

It can be observed that from the outset of the estimation process, despite the straight-line path, the estimated trajectory begins to deviate from the correct trajectory. This phenomenon is referred to as drift, and it occurs as a result of the accumulation of position estimation errors. It is important to provide a definition of error. Within the framework of the examined algorithm, error is delineated as the disparity in distance between points in a two-dimensional space employing a Cartesian coordinate system. It can be described as follows:

(1) $$E_{i}=\sqrt{{\left(x_{i}-x_{i-1}\right)}^{2}+{\left(y_{i}-y_{i-1}\right)}^{2}}$$

where

*E_i_*—one step estimation error;

*x_i_*—first coordinate in the Cartesian coordinate system at time “*i*”;

*y_i_*—second coordinate in the Cartesian coordinate system at time “*i*”.

The drift is calculated as the sum of the single-step estimation errors. This relationship can be described by the following formula:

(2) $$D=\sum_{i=0}^{N}E_{i}$$

where

*D*—drift;

*N*—number of sequences processed.

**Figure 12:**
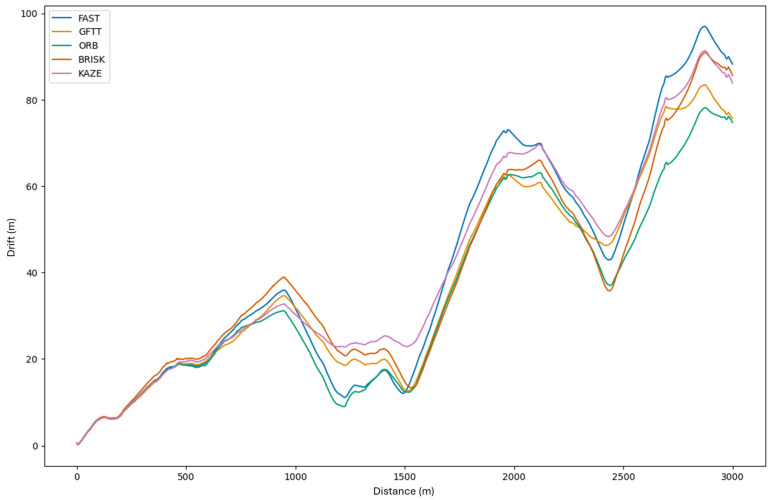
Dependence of drift on subsequent processed frames (distance) in the implemented algorithm for the tested methods.

Initially, the trajectories exhibit minimal error, to the extent that the discrepancy is imperceptible. As the number of frames processed in the sequence increases, the discrepancy becomes evident at subsequent positions. As demonstrated in the example of the last position, the discrepancy is substantial. However, it is worth noting that the estimated trajectory closely resembles the original path. As a means of facilitating an in-depth analysis of the drift values at individual moments in the image sequence processing, Figure 12 presents a graph that illustrates the drift for successive pairs of stereo image frames. The analysis of this graph can be based on checking which method produces the smallest drift at the last point of the estimated path. The drift achieved for each method at the conclusion of the estimation process is outlined below:

- FAST: D = 88.25 m;
- GFTT: D = 75.66 m;
- ORB: D = 74.76 m;
- BRISK: D = 85.76 m;
- KAZE: D = 83.86 m.

The drift values, estimated based on the implemented stereo vision odometry algorithm for each method, are low, below 5%, for a 2299 m long route.

**Figure 13:**
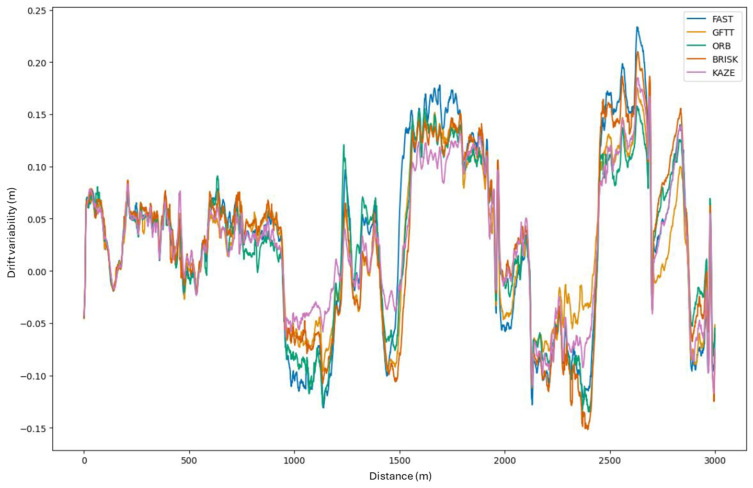
Drift variability from subsequent processed frames in the implemented algorithm for the tested methods.

The analysis of the graph is best based on examining how each variable varies from one frame to the next. This is because changes in direction of the estimated trajectory cause increases and decreases. Consequently, in the method that has been presented, the variable that undergoes the most rapid change has the greatest drift in a given section. In order to illustrate the variability, Figure 13 presents a processed graph showing the variability of drift for successive frames of the recorded route. The GFTT method is thus considered to be the least susceptible to changes in the direction of vehicle movement. The drift for this method is subject to the most negligible change. Conversely, the FAST method facilitates the observation of the most rapid changes in drift in individual sections of the graph. In accordance with predictions, the ORB method, which is based on FAST, is characterized by similar drift variability in many places, despite modifications to FAST. The result for the KAZE method is also of interest, as it suggests that it is resistant to changes in direction in the algorithm used. This finding aligns with the extant literature, which highlights its resistance to rotation.

**Figure 14:**
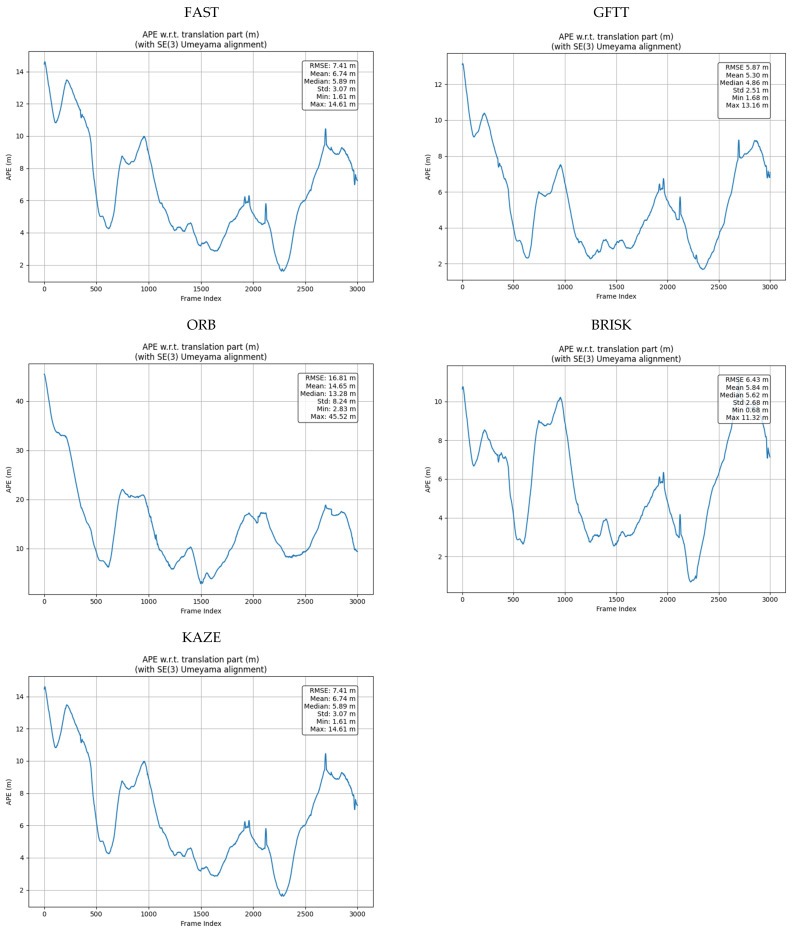
Pose error against ground truth for the in the implemented algorithm for the tested methods.

In addition, in Figure 14 we present the pose error against ground truth for the investigated algorithms. The figure shows the APE (Absolute Pose Error) values relative to the translational portion of the trajectory, which is expressed in meters. This error represents the difference between the estimated and reference path of the system. The data were presented after the Umeyama fit was applied in the Lie SE(3) group space (Special Euclidean group in 3D).

#### 3.2.2. Processing Time

**Figure 15:**
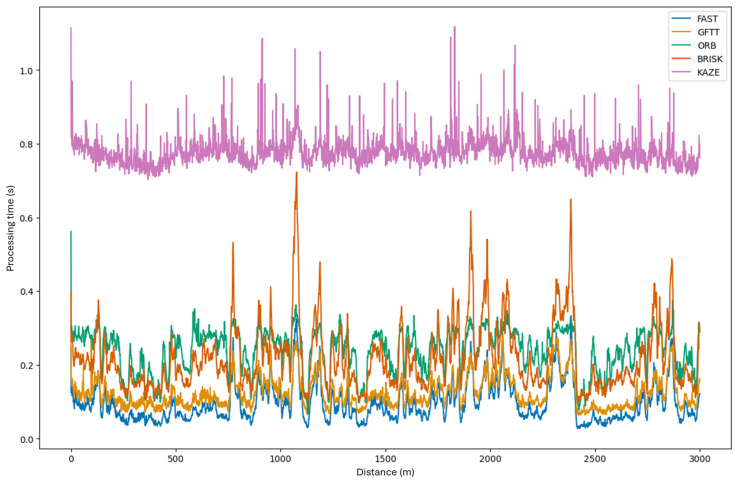
Dependence of the processing time of the stereovision odometry algorithm on subsequent frames of the tested sequence for the tested methods.

In consideration of the algorithm’s implementation elucidated in preceding chapters, it is imperative to assess the processing time of the subsequent step estimation loop. For this purpose, data was collected from the testing of a selected dataset with a selected sequence following the route from the previous study. The results are presented in Figure 15 as a graph showing the dependence of the processing time of each image pair in the sequence as a function of frame number versus frame number. A detailed examination of the graph reveals that for the selected dataset, the KAZE method does not align with the established processing time requirements. The data contained within the image sequence have been sampled at a frequency of 2.5 Hz. This results in an upper time limit of 400 milliseconds for the processing time of each image pair. It is worth noting, however, that this method corroborates the conclusions of the preceding study. In instances where frames depict a sudden change in direction (i.e., turns) within the scene, there is no observed increase in processing time. However, as demonstrated in the graph, this phenomenon occurs for all other methods. The phenomenon is characterized by an increase in values, indicative of the temporal extension observed for particular frame numbers. This effect is most pronounced in the BRISK method.

## 4. Conclusions

A study was conducted with the objective of comparing different keypoint selection methods and assessing their impact on trajectory estimation results in a stereo odometry algorithm. The objective of the study was to ascertain which of these methods provides the most accurate results in a real-world environment while meeting the required constraints. These constraints encompass the algorithm’s real-time processing requirements and the minimization of resource consumption by reducing the number of processed points.

The impact of the scene in the stereovision images on the selected keypoints was analyzed, as well as the processing time. In the course of the study of keypoint selection methods, the dependence of the number of detected keypoints on image resolution was also examined. The impact of potential interference on the aforementioned methods was also examined. In the following analysis, the impact of keypoint selection methods on the process of matching keypoints between pairs of stereovision images was investigated.

The present analysis examined the quality of the selected keypoints by the given methods, as measured by the ratio of matched keypoints to detected keypoints. The dependence of processing time and the number of found keypoints for various typical scenes in the studied set was also analyzed. The subsequent stage of the research was to conduct a route estimation analysis, which verified the match between the estimated route and the actual travel. The drift of the estimated route and the algorithm’s processing time were determined.

The study revealed a strong correlation between the number of keypoints and processing time, which differed depending on the scene and method used. With the exception of KAZE, all methods showed increased processing time with more keypoints. FAST was the most efficient method, quickly detecting many points. However, an excessive number of keypoints is not ideal for embedded stereo odometry, since irrelevant features can impede processing and diminish the quality of matches.

The analysis shows that while lower resolution increases the number of detected keypoints, higher resolution does not significantly improve estimation accuracy and only prolongs processing time. Nonetheless, resolution must remain sufficiently high to ensure accurate depth estimation from stereo images. As resolution decreases, all methods detect fewer keypoints in a proportional manner.

The impact of noise on stereo images shows that most methods are sensitive to pixel-level distortions, such as blur or white noise. These distortions can cause errors in trajectory estimation due to triangulation issues. Global intensity changes have little effect, but localized distortions such as overexposure are more disruptive. Of the methods tested, GFTT consistently maintained a stable number of detections under noisy conditions.

This study was conducted in order to test various methods of selecting points, in addition to descriptors that would be used for the purpose of keypoint matching between stereovision images. The results of the tests allowed the following conclusions to be drawn. The quality of keypoints, defined as uniqueness in the image, is a crucial aspect for subsequent keypoint matching. The uniqueness was determined by the ratio of keypoints matched to those detected in the images. The GFTT method was found to be the most effective in this regard. The processing time of the keypoint matching algorithm confirms the importance of keypoint quality for matching results. The FAST method is the most efficient for keypoint detection; however, it is important to note that keypoints require a significantly longer time to match in stereovision images.

The final part of the study aimed to examine the direct impact of keypoint selection methods on the quality of trajectory estimation. The trajectory estimation results for each of the tested methods were then compared with the original trajectory. The resulting trajectory shapes for each method were found to be similar. Drift, defined as the discrepancy between the estimated and actual trajectories, is a pivotal factor in the interpretation of these results. The analyzed route contains numerous orientation changes on the map presented in a Cartesian coordinate system. Consequently, the drift variability for subsequent frames in the sequence was examined. The findings derived from these observations demonstrate that the KAZE and GFTT methods exhibit optimal performance in the context of orientation changes. The FAST and ORB methods demonstrate the poorest performance, exhibiting the most significant drift variability.

Time analyses of the impact of the tested methods on the stereovision odometry algorithm allow us to rule out the KAZE method as failing to meet the processing time assumptions. In this particular context, the FAST and GFTT methods have been shown to be the most effective.

The findings of the research indicate that the GFTT method possesses optimal characteristics for incorporation into a stereo vision odometry algorithm, particularly in the context of trajectory estimation under real-world conditions.

## Figures and Tables

**Table 1:** Selected parameter of detector and detector plus descriptor methods.

FAST	GFTT	ORB	BRISK	KAZE
threshold: 10	qualityLevel: 0.01	nfeatures: 10,000	thresh: 30	threshold: 0.001
nonmaxSuppression: true	minDistance: 1	scaleFactor: 1.2	octaves: 3	nOctaves: 4
type: TYPE_9_16	maxCorners: 10,000	nlevels: 8	patternScale: 1.0	nOctaveLayers: 4
	useHarrisDetector: false	fastThreshold: 20		diffusivity: DIFF_PM_G2
		edgeThreshold: 31		

**Table 2:** Summary of the number of detected keypoints for the tested methods in the image from the left camera.

Scene	FAST	GFTT	ORB	BRISK	KAZE
Crossroad	6197	3744	8482	3605	2517
Traffic	4576	3030	7666	2938	1701
Straight road	6368	2822	6665	2302	1363
Buildings	3865	1327	3880	1304	939
Empty road	6803	3781	6900	2174	1415
Houses	9416	6294	8954	4962	2656

**Table 3:** Summary of the number of detected keypoints for the tested methods in the image from the right camera.

Scene	FAST	GFTT	ORB	BRISK	KAZE
Crossroad	5089	2998	8088	2925	2453
Traffic	3650	2637	6685	2402	1531
Straight road	4668	2369	5486	1798	1233
Buildings	2790	1125	3554	1125	866
Empty road	5388	3111	5981	1833	1364
Houses	8307	5543	8820	4471	2697

**Table 4:** Results of detection of keypoints for the tested methods when changing the resolution of the tested image. Resolution (px, px).

Method	(3840, 2160)	(2560, 1440)	(1920, 1080)	(1280, 720)	(800, 600)	(640, 480)
FAST	58,131	33,222	21,349	11,343	6678	4535
GFTT	34,014	18,925	11,221	6944	4372	2808
ORB	71,793	40,836	25,627	12,947	7199	4740
BRISK	18,512	12,417	8287	4977	2960	2016
KAZE	9496	4387	2540	1182	639	463

**Table 5:** Keypoints detected by the analyzed methods.

Method	Original	Noisy	Blurred	Distorted	Brightened	Darkened
FAST	144,746	370,041	1254	106,233	40,136	168,391
GFTT	77,481	293,581	57,028	67,257	77,805	93,113
ORB	149,905	179,624	4795	140,178	17,616	167,596
BRISK	49,014	375,369	1245	37,684	2480	67,745
KAZE	19,250	17,252	4062	17,571	1097	25,107

**Table 6:** Summary of matching results for the tested methods with the ORB descriptor and the ratio of the number of matched points.

Method	Right Image	Left Image	Matching	% Matched Keypoints	Inlier	Outliers	Precision
FAST	3904	5159	2257	57.8%	1124	1133	0.61
GFTT	1996	2290	1228	61.5%	704	524	0.41
ORB	2967	2977	1719	57.9%	995	742	0.66
BRISK	1751	2205	1012	57.8%	519	493	0.58
KAZE	1149	1282	675	58.7%	375	300	1.0

**Table 7:** Keypoints detection time and keypoints matching time with the ORB descriptor.

	FAST	GFTT	ORB	BRISK	KAZE
Time 1 (ms)	3.00	43.00	14.00	36.03	427.05
No. of keypoints	3904	1996	2967	1751	1149
Keypoints/Time ratio	1299.57	46.42	211.90	48.60	2.69
Matches	2257	1228	1719	1012	675
Time 2 (ms)	11.00	5.00	9.00	8.01	5.00
Matches/Time ratio	354.97	399.40	329.71	218.70	229.77
Total Time (ms)	14.00	48.00	23.00	44.04	432.05
CPU Usage (%)	12.5	18	15.2	17.8	25
Memory Usage (MB)	62	102	86	110	210

## Data Availability

Publicly available datasets were analyzed in this study. The dataset can be found here: https://www.cvlibs.net/datasets/kitti/ (accessed on 31 August 2025).

## References

[1] Balntas V., Lenc K., Vedaldi A., Mikolajczyk K. (2017). HPatches: A Benchmark and Evaluation of Handcrafted and Learned Local Descriptors. Proceedings of the 2017 IEEE Conference on Computer Vision and Pattern Recognition (CVPR).

[2] Fan P., Men A., Chen M., Yang B. Color-SURF: A surf descriptor with local kernel color histograms. Proceedings of the 2009 IEEE International Conference on Network Infrastructure and Digital Content.

[3] Vailaya A., Figueiredo M.A.T., Jain A.K., Zhang H.-J. (2001). Image classification for content-based indexing. IEEE Trans. Image Process..

[4] Ferrari V., Tuytelaars T., Van Gool L., Ponce J., Hebert M., Schmid C., Zisserman A. (2006). Simultaneous Object Recognition and Segmentation by Image Exploration. Toward Category-Level Object Recognition.

[5] Fan B., Kong Q., Wang X., Wang Z., Xiang S., Pan C., Fua P. (2019). A performance evaluation of local features for image-based 3D reconstruction. IEEE Trans. Image Process..

[6] Jakubović A., Velagić J. Image Feature Matching and Object Detection Using Brute-Force Matchers. Proceedings of the 2018 International Symposium ELMAR.

[7] Sakai Y., Oda T., Ikeda M., Barolli L. An Object Tracking System Based on SIFT and SURF Feature Extraction Methods. Proceedings of the 2015 18th International Conference on Network-Based Information Systems.

[8] Cao W., Ling Q., Li F., Zheng Q., Wang S. A keypoint-based fast object tracking algorithm. Proceedings of the 2016 35th Chinese Control Conference (CCC).

[9] Ignat A., Păvăloi I. (2021). Keypoint Selection Algorithm for Palmprint Recognition with SURF. Procedia Comput. Sci..

[10] Dixit D., Parashar S., Gondalia A., Sengupta A., Sivagami M. Facial Identification using Haar Cascading with BRISK. Proceedings of the 2020 International Conference on Emerging Trends in Information Technology and Engineering (ic-ETITE).

[11] Sikder J., Datta N., Tripura S., Das U.K. Emotion, Age and Gender Recognition using SURF, BRISK, M-SVM and Modified CNN. Proceedings of the 2022 International Conference on Electrical, Computer and Energy Technologies (ICECET).

[12] Pala S., Jayan S., Kurup D.G. (2020). An accurate UWB based localization system using modified leading edge detection algorithm. Ad Hoc Netw..

[13] Merzlyakov A., Macenski S. A Comparison of Modern General-Purpose Visual SLAM Approaches. Proceedings of the 2021 IEEE/RSJ International Conference on Intelligent Robots and Systems (IROS).

[14] Barros A.M., Michel M., Moline Y., Corre G., Carrel F. (2022). A Comprehensive survey of visual SLAM algorithms. Robotics.

[15] Campos C., Elvira R., Rodríguez J.J.G., Montiel J.M.M., Tardós J.D. (2021). ORB-SLAM3: An Accurate Open-Source Library for Visual, Visual–Inertial, and Multimap SLAM. IEEE Trans. Robot..

[16] Klein G., Murray D. Parallel Tracking and Mapping for Small AR Workspaces. Proceedings of the 2007 6th IEEE and ACM International Symposium on Mixed and Augmented Reality.

[17] Yu H., Wang Q., Yan C., Feng Y., Sun Y., Li L. (2024). DLD-SLAM: RGB-D Visual Simultaneous Localisation and Mapping in Indoor Dynamic Environments Based on Deep Learning. Remote Sens..

[18] Thrun S., Montemerlo M. (2006). The Graph SLAM Algorithm with Applications to Large-Scale Mapping of Urban Structures. Int. J. Robot. Res..

[19] Lowe D.G. (2004). Distinctive Image Features from Scale-Invariant Keypoints. Int. J. Comput..

[20] Rublee E., Rabaud V., Konolige K., Bradski G. ORB: An efficient alternative to SIFT or SURF. Proceedings of the 2011 International Conference on Computer Vision.

[21] Leutenegger S., Chli M., Siegwart R.Y. BRISK: Binary Robust invariant scalable keypoints. Proceedings of the 2011 International Conference on Computer Vision.

[22] Alcantarilla P.F., Bartoli A., Davison A.J., Fitzgibbon A., Lazebnik S., Perona P., Sato Y., Schmid C. (2012). KAZE Features. Computer Vision—ECCV 2012.

[23] Yi K.M., Trulls Fortuny E., Lepetit V., Fua P. LIFT: Learned Invariant Feature Transform. Proceedings of the European Conference on Computer Vision (ECCV).

[24] DeTone D., Malisiewicz T., Rabinovich A. SuperPoint: Self-Supervised Interest Point Detection and Description. Proceedings of the 2018 IEEE/CVF Conference on Computer Vision and Pattern Recognition Workshops (CVPRW).

[25] Ono Y., Trulls E., Fua P., Yi K. (2018). LF-Net: Learning Local Features from Images. arXiv.

[26] Dusmanu M., Rocco I., Pajdla T., Pollefeys M., Sivic J., Torii A., Sattler T. D2-Net: A Trainable CNN for Joint Description and Detection of Local Features. Proceedings of the 2019 IEEE/CVF Conference on Computer Vision and Pattern Recognition (CVPR).

[27] Revaud J., Weinzaepfel P., De Souza C., Humenberger M. (2019). R2D2: Repeatable and reliable detector and descriptor. Proceedings of the 33rd International Conference on Neural Information Processing Systems.

[28] Bresson G., Aufrère R., Chapuis R. Improving SLAM with Drift Integration. Proceedings of the IEEE 18th International Conference on Intelligent Transportation Systems.

[29] Geiger A., Lenz P., Stiller C., Urtasun R. (2013). Vision meets robotics: The KITTI dataset. Int. J. Robot. Res..

[30] Rosten E., Drummond T. Machine Learning for High-Speed Corner Detection. Proceedings of the European Conference on Computer Vision.

[31] Shi J., Tomasi C. Good features to track. Proceedings of the 1994 Proceedings of IEEE Conference on Computer Vision and Pattern Recognition.

